# Dynamics of gene regulatory networks and their dependence on network topology and quantitative parameters – the case of phage *λ*

**DOI:** 10.1186/s12859-019-2909-z

**Published:** 2019-05-31

**Authors:** Dace Ruklisa, Alvis Brazma, Karlis Cerans, Thomas Schlitt, Juris Viksna

**Affiliations:** 1 Newnham College, University of Cambridge, Sidgwick Avenue, Cambridge, CB3 9DF UK; 2 European Molecular Biology Laboratory, European Bioinformatics Institute, EMBL-EBI, Wellcome Genome Campus, Hinxton, CB10 1SD UK; 3 Institute of Mathematics and Computer Science, University of Latvia, Rainis blvd. 29, Riga, LV 1459 Latvia; 4 Division of Molecular Neuroscience, Faculty of Psychology, University of Basel, Basel, 4055 Switzerland

**Keywords:** Gene regulatory networks, Hybrid systems, Phage *λ*, Stable behaviours, Model validation

## Abstract

**Background:**

Gene regulatory networks can be modelled in various ways depending on the level of detail required and biological questions addressed. One of the earliest formalisms used for modeling is a Boolean network, although these models cannot describe most temporal aspects of a biological system. Differential equation models have also been used to model gene regulatory networks, but these frameworks tend to be too detailed for large models and many quantitative parameters might not be deducible in practice. Hybrid models bridge the gap between these two model classes – these are useful when concentration changes are important while the information about precise concentrations and binding site affinities is partial.

**Results:**

In this paper we study the stable behaviours of phage *λ* via a hybrid system based model. We identify wild type and mutant behaviours that arise for various orderings of binding site affinities. We propose experiments for detecting these behaviours: we suggest several ways of altering binding affinities with either mutations or genome rearrangements to achieve modified behaviours. The feasibility of these experiments is assessed. The interplay between the qualitative aspects of a network, e.g. network topology, and quantitative parameters, e.g. growth and degradation rates of proteins, is demonstrated. We also provide a software for exploring all feasible states of a hybrid system model and identifying all attractors.

**Conclusions:**

The behaviours of phage *λ* are determined mainly by the topology of this network and by the mutual order of binding affinities. Exact affinities and growth and degradation rates of proteins fine tune the system. We show that only two stable behaviours are possible for phage *λ* if the main constraints of *λ* switch are preserved – these behaviours correspond to lysis and lysogeny. We identify several variants of both lysis and lysogeny – one wild type and one modified behaviour for each. We elucidate the necessary constraints for binding site affinities to achieve both wild type lysis and lysogeny. Our software is applicable to a wide range of biological models described as a hybrid system.

**Electronic supplementary material:**

The online version of this article (10.1186/s12859-019-2909-z) contains supplementary material, which is available to authorized users.

## Background

Gene regulatory networks can be modelled in various ways that range from listing the building blocks of a network to detailed simulations in time by differential equations [1]. Picking the right modelling approach is not trivial; the choice is often determined by the intended scope of a model, the available data, e.g. their detail, quality and ‘completeness’, and also by the availability of a modelling software.

One of the earliest formalisms employed for the modelling of gene regulatory networks is a Boolean network [2, 3]. Being comparatively simple, these models allow to identify all possible behaviours of a system. Boolean networks have been used to study stable behaviours of phage *λ* by systematically exploring possible states of this biological system [4]. However, these models have limitations for describing the temporal aspects of behaviours because concentrations are not explicitly modelled. Whenever the response of a gene regulatory network differs depending on the concentration of a biological entity (such as a transcription factor or a signalling molecule) it becomes necessary to find various artificial ways to incorporate this behaviour in the model. To overcome this hurdle, several generalisations of Boolean networks have been proposed [5–7]. Boolean networks have been used in modelling the differentiation of myeloid and lymphoid cell lines and reprogramming between cell types that can induced by specific transcription factors [8]. In this study, the effects of various knockdowns were simulated and compared with experimental data. A Boolean dynamic model was built to study combinatorial interventions that could potentially inhibit epithelial-to-mesenchymal transition and thus suppress the invasive properties of cancer cells and reduce the potential for metastases [9]. The effects of perturbations were explored, after at least one element of the network was either inactivated or consistently activated. The predicted perturbation results were validated with siRNA experiments and there was a sufficient overlap between in silico and in vivo results.

Differential equation models have also been used to model gene regulatory networks, although these frameworks tend to be too detailed for large models. The parameters characterising dynamics cannot be easily obtained from experimental data. For example, precise binding site affinities under different biological conditions and temperatures are difficult to determine. The analysis of such a model can become intractable for a large number of quantitative parameters. Thus, hybrid models are a welcome alternative when concentration changes have to be modelled while the information about concentrations and binding site affinities is partial. One such hybrid model is based on the hybrid system formalism [10]. It has been shown that this type of model is adequate for describing and simulating biological networks [11]. To facilitate and simplify the analysis of hybrid system models many authors have sought to define restricted classes of models – these have been used to describe a variety of biological systems [12–16]. For example, *Drosophila* circadian cycle has been captured in such a model [17]. See also models of cardiac cells and bone cells respectively [18, 19]. Other models of this type have been used to analyse the stability of cyclic behaviours [6, 7], and have been instrumental in demonstrating the stability of the regulatory circuits of phage *λ* [7].

In this paper we present and describe in detail a novel model of phage *λ*. We carry out an exhaustive analysis of possible model behaviours and identify different stable behaviours (attractors). We prove that phage *λ* can exhibit only two steady states due to the way in which its regulatory elements work and cooperate. We confirm that all attractors of the model correspond to biologically known behaviours, namely lysis and lysogeny. However, either of these steady states can be altered when the relative order of critical binding affinities is changed. We also describe the characteristics of these altered states. Next, we determine sufficient conditions for binding site affinities for achieving a wild-type behaviour of phage *λ*. Finally, we propose experimentally testable hypotheses about the behaviour of phage *λ* under different regulatory constraints. We suggest several experiments that could verify our findings.

We employ the Hybrid System Model (HSM) approach – it allows for a broad range of models comprising both discrete and continuous variables. This framework offers sufficient flexibility in describing a biological system as it does not require complete knowledge of binding site affinities and the shapes of growth and degradation functions. This modelling technique is a generalisation of the Finite State Linear Model (FSLM) which the authors have developed previously [1, 20–22]. Within the HSM framework we drop the assumption that concentrations change according to linear functions; instead we allow the changes to be monotonous. The HSM framework was introduced in [23] and the corresponding mathematical formalism was described in detail in [24] alongside the algorithms performing automated analysis of all possible states of a HSM model.

In this paper we outline the procedure of finding attractors when a partial ordering of binding affinities is known. We are building on our earlier work in which we proposed an algorithm for analysing all system behaviours when for each protein a full linear ordering of binding affinities was known [23].

We also provide a software for analysing biological models described within the HSM framework. It explores all feasible states of a network and identifies all attractors. Our implementation classifies attractors according to their dependence on quantitative parameters, distinguishes the states and cycles in which it is possible to stay indefinitely, and provides descriptions of the dynamic behaviours within attractors. The tool is computationally fast, i.e. it completes analysis in a few seconds, and is suitable for gene regulatory networks having up to twenty genes.

Our phage *λ* model is different from the one used in [1, 20–22] as it explicitly models both *O*_*R*_ and *O*_*L*_ cascades of operators. This model was implicitly used in [23], but without a complete definition. Our model is more general than the one defined in [7]: it permits a broader range of growth and degradation functions and captures additional biological details.

**Fig. 1:**
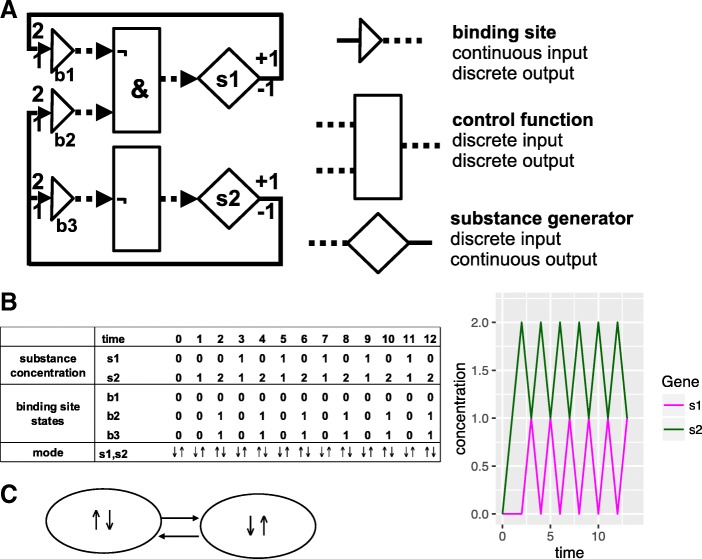
A small network with two genes. Gene **s1** has a negative feedback loop on itself, gene **s2** has a negative feedback loop on itself and a positive feedback on gene **s1**. **a** The network model. There are three binding sites, represented by triangles (**b1, b2, b3**), two control functions, represented by rectangles, and two substance generators, represented by diamonds (**s1, s2**). Discrete inputs and outputs correspond to dotted lines, while continuous inputs and outputs are represented by continuous lines. For each binding site, the association constant is given above and the dissociation constant below an incoming arrow. The substance generators **s1** and **s2** can either produce a substance or not – here linear growth and degradation rates are written above and below the output line. The binding sites can be unoccupied (‘0’ in the table) or occupied (‘1’ in the table). **b** A simulation run of the network. With association and dissociation constants given in (**a**) the network switches between two modes where either **s1** is active and **s2** inactive (*↑↓*) or vice versa (*↓↑*). **c** A graphical representation of the qualitative observational sequence describing the simulation run (**b**) of the network given in (**a**).

A HSM model involves both discrete and continuous components. Four elements can serve as building blocks for models: *substances*, *binding sites*, *control functions* and *substance generators*. *Substances* represent various types of molecules such as small molecules or proteins; their concentrations are modelled by continuous variables. The states of *binding sites* are modelled by discrete variables; a binding site can have two states, ‘on’ and ‘off’, or more than two states if several substances can bind to it. The state of a binding site depends on the concentration of a substance that can bind to it. The states of sites determine the expression levels of genes (the relationship between an expression level and binding site states is defined by a discrete *control function*, one for each gene; a finite number of expression levels is possible). *Substance generators* provide growth and degradation functions describing the changes of protein concentrations over time – these functions are monotonous. A simple HSM model with three binding sites and two genes is shown in Fig. 1.

## Methods

### A phage *λ* model

Phage *λ* has served as a model organism for a long time and its molecular biology is well understood by now [25]. The virus phage *λ* infects *Escherichia coli* cells. Upon the infection of a bacterial cell phage *λ* chooses one of the two modes of operation, *lysis* or *lysogeny* [26]. During *lysogeny* the phage DNA is integrated into the bacterial genome and the gene *cI* that encodes *repressor* protein is the only active phage gene. During *lysis* the phage DNA is excised, replicated, and new phage particles are produced. At the end of this process the bacterium is broken open (lysed) to release phage particles.

The molecular mechanism underlying the lysis-lysogeny decision is known as a *λ* switch; it involves several cascades of events and multiple genes [25]. In a nutshell, the state of a *λ* switch is determined by the concentrations of two proteins, namely *repressor* (encoded by the *cI* gene) and *Cro* (encoded by the *cro* gene). (Henceforward the names starting with capital letters will refer to proteins, while the names starting with lower case letters will denote genes.)

**Fig. 2:**
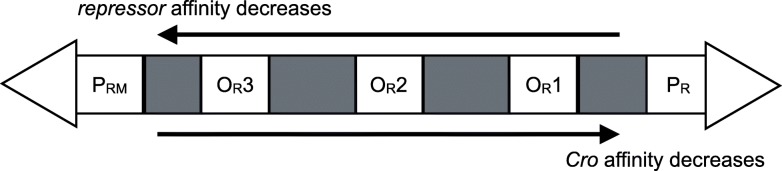
The main gene regulatory component of phage *λ*. Operator sites *O*_*R*_1, *O*_*R*_2 and *O*_*R*_3 are located between the promoters *P*_*RM*_ and *P*_*R*_ on the phage *λ* genome

The switching of phage *λ* between various behaviours is mainly implemented by the operator sites *O*_*R*_1, *O*_*R*_2 and *O*_*R*_3. These sites are located between two *λ* promoters: *P*_*R*_, which controls the transcription of the *cro* gene, and *P*_*RM*_ controlling the transcription of the *cI* gene which encodes the *repressor* (Fig. 2) [26–29]. All three sites can be bound by either *Cro* or *λrepressor* albeit with opposite orderings of the strengths of binding affinities. The *λrepressor* binds to the operator site *O*_*R*_3 when in very high concentration (affinity is low). When *repressor* is bound to *O*_*R*_3 it abolishes the transcription of the *cI* gene thereby establishing a negative feedback loop on itself. At much lower concentrations *λrepressor* binds to *O*_*R*_1 and thus inactivates the transcription of *cro*. The affinity order for *Cro* and these sites is opposite. At low concentrations *Cro* can bind *O*_*R*_3, thus inactivating the transcription from the promoter *P*_*RM*_ and the *cI* gene. At higher concentrations *Cro* occupies *O*_*R*_2 and then *O*_*R*_1, thus inactivating the transcription from *P*_*R*_ and its own gene [25, 25, 26, 29]. The remaining genes on the *λ* genome mainly encode the proteins that form a phage particle and enzymes necessary for integration into and excision from the host cell genome. Many phage *λ* genes are organised in operons, i.e. one mRNA molecule actually encodes several proteins. The terminator sites between genes determine whether a RNA polymerase will move beyond a terminator site or not and whether long or short transcripts will be made. This read-through is dependent on the presence of the anti-terminator protein *N*.

**Fig. 3:**
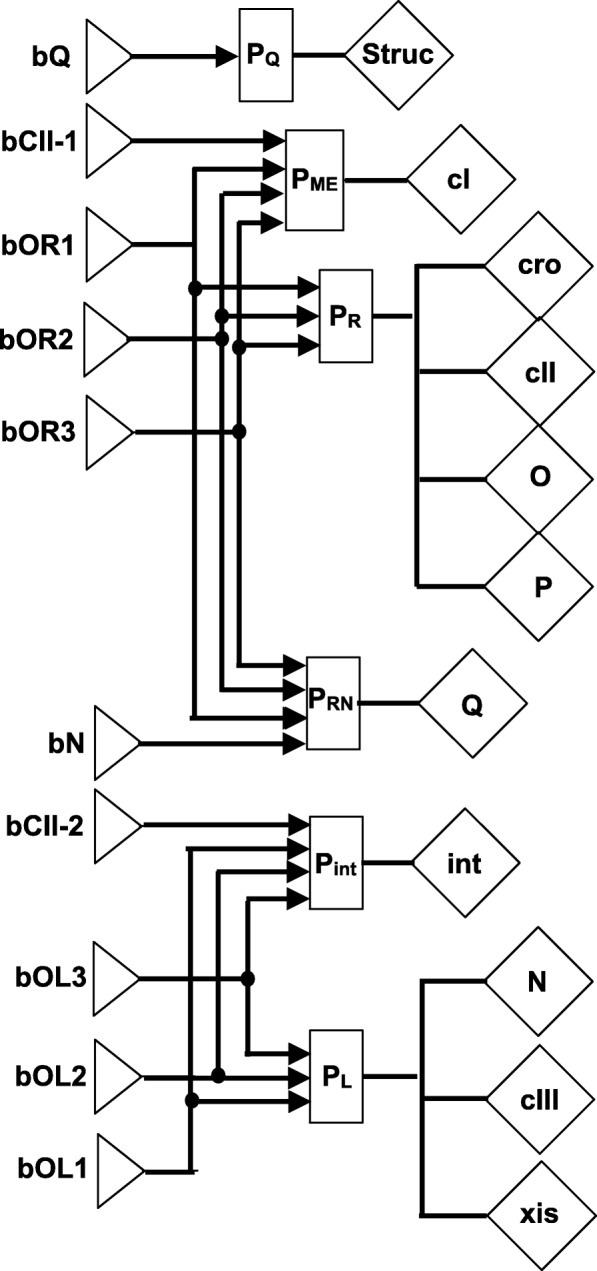
A phage *λ* model. The model is defined by specifying genes and binding sites and their mutual relationships – these entities uniquely determine the components of a biological system that are captured by a HSM model. Individual parts of the model are described in Materials and Methods section ‘A phage *λ* model’

Here, we describe a HSM model for phage *λ* that captures essential biological aspects (Fig. 3). It is a novel model that includes additional elements in comparison with our earlier models used in [1, 20–22]. In particular, operator sites *O*_*R*_1,*O*_*R*_2,*O*_*R*_3,*O*_*L*_1,*O*_*L*_2 and *O*_*L*_3 are modelled, and each of them can potentially have distinct binding affinities for *repressor* and *Cro*. To make the model diagram more legible, we do not draw the lines between substance generators (depicted by diamonds) and the binding sites they affect (depicted by triangles).

The model consists of ten binding sites, six control functions (*P*_*Q*_,*P*_*ME*_,*P*_*R*_,*P*_*RN*_,*P*_*int*_,*P*_*L*_) and eleven substance generators (**cI, cro, cII, O, P, Q, int, N, cIII, xis** and **struc** where **struc** represents the genes encoding structural proteins) (Fig. 3). We use bold face to denote model elements and to distinguish them from biological entities; the mapping of names is shown in Table 1. There are two sets of three binding sites **bOR1, bOR2, bOR3** and **bOL1, bOL2, bOL3** where each binding site can be in three different states: unbound, bound by **Cro**, or bound by **repressor**. The **repressor** binding affinities form a descending order (i.e. association constants increase) for the sites **bOR1, bOR2, bOR3** and also for the sites **bOL1, bOL2, bOL3** (both orderings are separate). For **Cro** the affinities to these binding sites have a reverse order of strength in comparison with **repressor**. The remaining binding sites (**bQ, bCII-1, bCII-2** and **bN**) can only be bound by one protein each and therefore have two states only (bound and unbound).

**Table 1:** Summary of the promoters and terminators of phage *λ* and the corresponding components of our model

Phage *λ* entity	Model component
*P*_*E*_	*P*_*ME*_
*P*_*int*_	*P*_*int*_
*P*_*L*_	*P*_*L*_
*P*_*M*_	*P*_*ME*_
*P*_*R*_	*P*_*R*_
$P_{R6^{\prime }}$	*P*_*Q*_
*t*_*R*1_,*t*_*L*1_,*t*_*L*2_	–
*t*_*R*2_	*P*_*RN*_

The biological entities that are directly represented in our model map to components depicted in Fig. 3

The terminators are not represented in the model directly. Instead the function that determines the expression of a gene incorporates conditions imposed by terminators. As we do not model different growth and degradation rates and consider only two states for each substance generator (‘on’ or ‘off’), the terminators affecting the precise level of expression were not modelled at all. It means that only *t*_*R*1_ was included in the model.

The *λ*-switch is implemented in the model by coupling the binding sites **bOR1, bOR2** and **bOR3** with the control functions *P*_*R*_,*P*_*RN*_ and *P*_*ME*_. *P*_*ME*_ affects the expression of **cI**, whereas *P*_*R*_ controls the expressions of **cro, cII, O** and **P**. To model the behaviour of the terminator *t*_*R*2_, we introduced the promoter *P*_*RN*_. *P*_*RN*_ is active in the presence of **N** (i.e. when **bN** is in the ‘bound’ state) just like *P*_*R*_; in the absence of **N** it becomes inactive. Thus the expression of **Q** is determined by the concentrations of **Cro, repressor** and **N**. *P*_*ME*_ receives input from binding sites **bOR1, bOR2, bOR3** and **bcII-1** and ensures that the activity of **cI** depends on the concentrations of **repressor** and **Cro**, and can also be triggered by **CII** reaching a certain threshold. The binding sites **bOL1, bOL2** and **bOL3** affect *P*_*L*_ that in turn influences **N, cIII** and **xis**. The degradation of **repressor** that is triggered by stress response proteins is not explicitly incorporated in this model, but can be mimicked by choosing suitable starting concentrations.

A full specification of our phage *λ* model is included in Additional file 1 (model description and constraints for binding site threshold orderings).

### Qualitative analysis of a HSM model

What happens when we run a simulation of, for example, a phage *λ* model? At the beginning the binding site states will switch to states compatible with the initial protein concentrations. The binding site states uniquely determine the gene expression levels via control functions. All expression levels will remain unchanged until the concentration of some protein reaches a threshold for a binding site that in turn triggers a change in the state of this site. It can happen when a protein concentration grows above an association constant or falls below a dissociation constant. As long as no binding site state is altered the protein concentrations will be either increasing or decreasing monotonously. Therefore we can unambiguously recreate the current state of the modelled biological system by noting the states of all binding sites and the time when the last switch of a binding site state took place and all concentrations at that point.

We can perform a qualitative analysis of behaviours of a HSM model by studying the states through which the model behaviour evolves and by dropping the times of change of binding site states. In this section we introduce a new method of qualitative analysis that does not require the knowledge of precise concentrations and of switching times.

We capture the current (qualitative) behavioural state by recording the states of all binding sites. We refer to such a record as a *mode*.

We call the actual course of the protein concentrations over time a *run*. Different *runs* can be generated by the same model by varying initial concentrations. A run is defined and uniquely determined by three components: the initial concentrations of all proteins, the sequence of modes through which the system evolves, and the sequence of time points when modes change.

It is straightforward to derive the information on growth and degradation directions from the sequence of modes in a run, because each mode uniquely determines the direction of change for each protein. A direction is coded by the following symbols: *↑* for increasing, → for unchanged and *↓* for decreasing. We will call a sequence of directions a *qualitative behaviour* as it omits both precise concentrations and growth and degradation rates.

In Fig. 1 we have depicted a small HSM network with two genes. In this case the qualitative behaviour for a simulation run can be recorded by a sequence of pairs of arrows. For example, the pair (*↑↑*) implies that the amounts of both proteins increase; (*↑↓*) shows that the s1’s concentration increases, while the s2’s decreases. A new pair has to be added to this sequence when the state of some binding site changes as this can subsequently alter the expression of a gene.

#### The characteristic graph for all model behaviours

We perform an analysis of the network dynamics by building a graph comprising all possible successions of modes. Previously we have proposed a method for exploring the graphs of all feasible states [24]. Such a graph captures all possible behaviours of a system that are compatible with given constraints for binding site affinity orderings. Here, we consider a simpler situation when we know the linear orderings of all binding affinities for each protein. We call the graph describing all behaviours permitted by such orderings a *characteristic graph*. In such a graph, an edge is drawn between two modes when the source mode can be followed by the target mode if the state of a certain binding site changes. Each characteristic graph is a directed graph – arrows indicate the order in which modes can occur. Each edge is augmented with a label stating which protein can trigger a change in a binding site state by reaching a concentration threshold. A triggering condition is denoted by an inequality between a protein concentration and a threshold (e.g. ‘Cro ≤ bOR2.dis’) and called a *transition guard*. In this graph we lose the information on protein concentrations and on the time it takes to move from one mode to the next. Constructing a characteristic graph involves identifying all possible successive modes by iterating through all binding site state changes that can occur in the current mode; for more details on the construction algorithm see [23]. For large networks enumerating all modes and traversing all transitions between them can be computationally expensive. However, the knowledge of relationships between association and dissociation constants can be used to reduce the number of modes drastically as the orderings of these constants provide many constraints for possible binding site state combinations. For example, for our phage *λ* model we know that the binding sites **bOR1, bOR2** and **bOR3** have a fixed order of affinities and that **bOR1** will always switch either before or simultaneously with **bOR2** when the concentration of **repressor** is increasing and the sites are vacant.

All sequences of modes that can occur in the simulations of a HSM model will be represented in a characteristic graph. However, not all paths in a characteristic graph will make biological sense and not all paths will be observable in simulations. When exploring possible binding site changes for a mode we are not checking whether there exists a set of initial concentrations that can lead to a specific transition given particular concentration change rates. Thus a characteristic graph provides a safe approximation of all simulation results that can be obtained from a HSM model by choosing various initial concentrations, initial binding site states and growth and degradation functions.

The software that constructs a characteristic graph for a biological model is included in Additional file 1. We used it for all analyses of this paper. Instructions for deploying it are incorporated there as well as an example of a complete ordering of all binding site thresholds.

#### Identification of stable behaviours

Analysis of the topology of a characteristic graph can reveal the number of different behaviours. We are interested in the parts of a characteristic graph that correspond to stable behaviours (*attractors*) of a biological system. A set of modes in a characteristic graph is an attractor if it permits a behaviour that stays exclusively within these modes for an infinite time. We are particularly interested in well-connected attractors: an attractor is well-connected if there is a path between each pair of modes within it.

The closest analogue to a HSM model attractor is a strongly connected component (SSC). A SSC is defined as a subgraph that contains a path between any two of its vertices. Any well-connected attractor lies within a SCC. There can, however, be SCCs that do not contain any attractors – this happens if no system behaviour can stay infinitely within this SCC. We recognise this situation by noticing a progress indicator (a protein). Such a protein either monotonously grows within all modes of a SSC but stays below at least one of its association thresholds or alternatively monotonously decreases within all modes of a SSC but stays above zero and at least one threshold for this protein. In this way we identify transitional components that certainly do not contain any attractors.

We find all candidate attractors in the characteristic graph of a HSM by identifying SCCs without any progress indicators. The lack of a progress indicator potentially allows an infinite behaviour to take place solely within this SSC. However, this condition alone does not guarantee that a particular run of a system will stay indefinitely within such a component or even that a run with this property will exist for a particular set of quantitative parameters. In addition we check whether a SSC is final, i.e. whether there is no edge leading outside of it.

We extracted and classified all stable states of a system by Perl scripts (see Additional file 1).

## Results

***Partially known binding affinities***

**Fig. 4:**
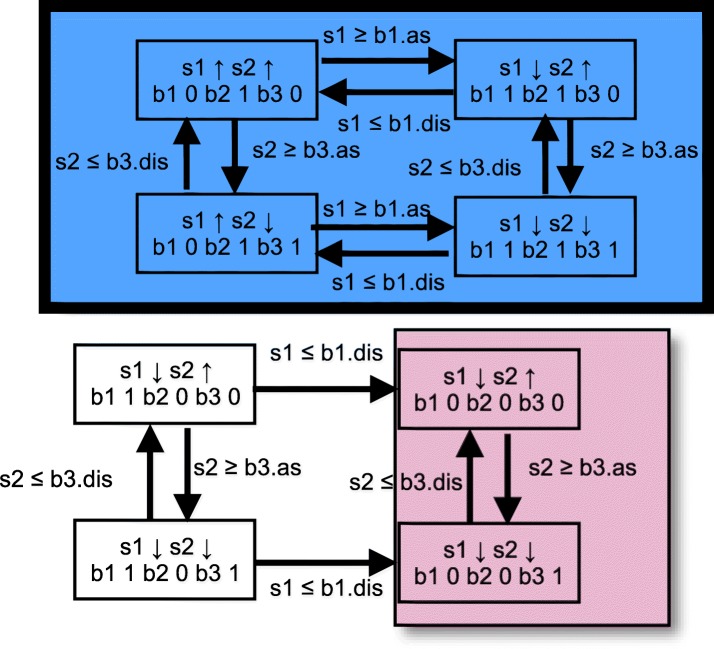
The characteristic graph of the small HSM network from Fig. 1 for the binding affinity ordering b2.dis < b3.dis < b3.as < b2.as for s2. Each mode in the graph can be identified by a unique binding site state combination; it is labeled by the concentration change direction for each protein. SSCs that contain attractors are either blue, with bold margins, or pink, with narrow margins. Pink attractors correspond to a single cycle whereas blue attractors comprise at least two cycles and at least one branching point leading into different cycles

When the precise ordering of binding site affinities is not known, then for a sufficiently small network we construct characteristic graphs for all possible binding site affinity orderings. We used a Perl script to enumerate all linear orderings that are compatible with given constraints for binding site affinities (see Additional file 1). For the network of Fig. 1 we built the graphs for the six orderings shown in Table 2. The first two yielded graphs with identical structure and attractors; the last two orderings also led to structurally equivalent graphs and identical attractors. Thus, we observed four different attractor structures for six threshold orderings (one of the characteristic graphs is depicted in Fig. 4). We detected three different attractors across all graphs. One of them was a simple auto-regulatory circuit with two vertices, while the other two were more complicated. The dynamics of the system was straightforward for each threshold ordering as it always led to a unique attractor, with the exception of the third ordering. The graph for the third ordering had two attractors (see Fig. 4), while the outcome of a behaviour was immediately determined by the initial state of binding sites.

**Table 2:** Possible orderings of association and dissociation thresholds for two binding sites when the same protein can bind both sites

Threshold orderings
b2.dis < b2.as < b3.dis < b3.as
b2.dis < b3.dis < b2.as < b3.as
b2.dis < b3.dis < b3.as < b2.as
b3.dis < b2.dis < b2.as < b3.as
b3.dis < b2.dis < b3.as < b2.as
b3.dis < b3.as < b2.dis < b2.as

Such orderings are considered for s2 when analysing the small network from Fig. 1

### Qualitative analysis of phage *λ* behaviours

We analysed the dynamics of the HSM model for phage *λ* under various constraints. We explored different orderings of binding site affinities for **Cro** or **repressor** to understand which conditions are critical for attaining a wild type behaviour and which aspects of the network can explain various mutant behaviours that have been observed experimentally. We built a characteristic graph for each combination of **Cro** and **repressor** threshold orderings that is consistent with the known affinity precedence within **bOR1, bOR2, bOR3** and within **bOL1, bOL2, bOL3**. The necessary affinity precedence was preserved for both **Cro** and **repressor**. The orderings for other proteins were assumed to be unique, because the binding concentration for **CII** and binding site **bCII-1** is known to be higher than for the site **bCII-2**. Furthermore, we assumed that the association and dissociation thresholds for a pair of a protein and a binding site are sufficiently close so that no other thresholds for the same protein can be situated between them. As an exception to the latter rule we allowed for two intermediate threshold orderings for **bOR2** and **bOL2** sites: bOR2.dis < bOL2.dis < bOR2.as < bOL2.as and bOL2.dis < bOR2.dis < bOL2.as < bOR2.as. These assumptions follow from the biology of phage *λ*, but might not always hold for other models. After taking into account these constraints we were left with 22×22=484 threshold orderings. In addition we tested the case when the affinities for **bOR2** and **bOL2** are equal for both **Cro** and **repressor** – 16 threshold orderings compatible with this and other constraints were considered. In total, 500 different threshold orderings were examined.

We built a characteristic graph for each of the 500 threshold orderings and detected SSCs in each graph. Surprisingly, there were exactly two attractors in each graph. We observed four different attractors across all threshold orderings. Two of them corresponded to the familiar lysis and lysogeny behaviours, while the remaining two were altered behaviours: one modified attractor was a mutated lysogeny behaviour and the other was a mutated lysis. (Several attractors had variations exhibiting some differences in binding site states, but no discrepancies in the qualitative behaviour – we do not distinguish between them and speak of four different attractor types instead of nine.) Thus, we found that there were no hidden attractors for phage *λ* beyond the lysis and lysogeny, while these two stable behaviours can vary phenotypically.

**Fig. 5:**
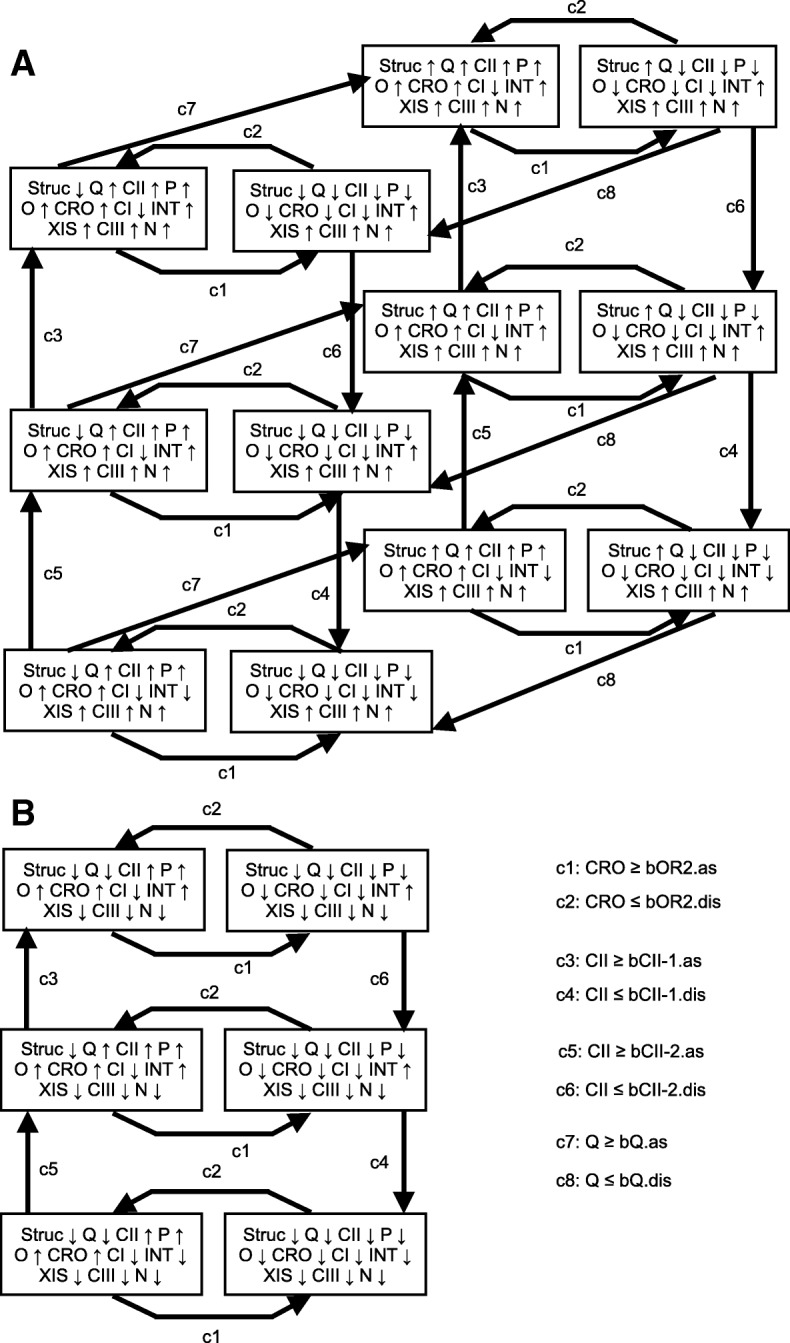
Phage *λ* attractors from the characteristic graph. **a** The wild type lysis attractor; **b** the modified lysis attractor. Note that binding site states are omitted from the mode labels, instead modes are marked solely by their qualitative behaviour, i.e. the growth and degradation indicators for all proteins

How do the wild type and modified lysis behaviours differ? The wild type lysis is represented in a characteristic graph by a SSC with twelve vertices and transitions triggered by three different proteins: **Cro, CII** and **Q** (see Fig. 5). The modified lysis behaviour corresponds to a SSC with six vertices; transitions with **Q** are absent from this behaviour (see Fig. 5). Both wild type and modified behaviours have characteristics in common: the concentration of **Cro** fluctuates between the association and dissociation thresholds of **bOR2** (an auto-regulatory circuit) and transitions with **CII** are similar.

What can be inferred about the wild type lysis behaviour from the characteristic graph? The concentration of **Cro** influences the concentrations of **CII** and **Q** via binding sites **bOR1, bOR2, bOR3**: when **Cro** is accumulating both **CII** and **Q** are increasing, but when **Cro** is being degraded also the concentrations of **CII** and **Q** are falling (see Fig. 5). Thus the dynamics of **Cro** determines how far the concentrations of **CII** and **Q** will rise or fall and also which of the transitions by **Q** and **CII** will actually be triggered. For example, if the upwards path between the dissociation and association thresholds of **Cro** and **bOR2** is traversed quickly, i.e. before **Q** can increase up to its binding affinity for the site **bQ**, the production of structural proteins will not be initiated. It means that the ratio of the growth rate of **Cro** to the growth rate of **Q** determines the observable behaviour within the lysis attractor. Similar observations can be made about the dependence of **CII** growth on the growth rate of **Cro**. We conclude that the wild type lysis behaviour is mainly determined by the network topology, however, there are some quantitative parameters that fine tune the observable behaviour. Most importantly, the ratios of the growth rates for **Cro, CII** and **Q** have to be such that the production of structural proteins is not inhibited, otherwise the wild type behaviour cannot occur.

The modified lysis behaviour is simpler than the wild type lysis (see Fig. 5). It is guaranteed that the structural proteins as well as proteins encoded by **Q, xis, cIII, N** will degrade to zero concentration in contrast to the wild type lysis. It means that a phage cannot be excised from the host genome and that several components will be lacking for assembling replicated phage particles. This attractor has transitions initiated by **Cro** and **CII** only. The auto-regulatory circuit of **Cro** controls the concentration of **CII**: when **Cro** increases also **CII** increases and the degradation of **Cro** implies the degradation of **CII**. Thus, the ratio of the growth rate of **Cro** to the growth rate of **CII** determines which thresholds can be reached by **CII** and consequently whether **Int** will be produced. Hence appropriate quantitative parameters are crucial to enable the integration of a phage genome into a host genome.

The main characteristics of the lysogeny behaviour are the fluctuation of **repressor** concentration between the dissociation and association thresholds of **bOR2** (an auto-regulatory circuit) and the gradual degradation of all other proteins to zero level. Within a modified lysogeny behaviour the products of **cIII, xis** and **N** are accumulating, while other proteins behave as in a wild type attractor. The modified behaviour implies that phage particles can be excised from the host genome and integrated into it, while replication will be difficult. The observable type of lysogeny behaviour is solely determined by the topology of the network; quantitative parameters do not play any role here.

### Model validation and sufficient conditions for altered behaviours

What are the necessary and sufficient conditions for obtaining a modified instead of a wild type attractor? To answer this, we determined shared behaviours for various threshold orderings. The analysis of characteristic graphs for 500 threshold orderings led us to a conclusion that both thresholds for *Cro* and *O*_*R*_2 have to be lower than the thresholds for *Cro* and *O*_*L*_2 to achieve a wild type lysis behaviour (interestingly enough, the mutual order of *repressor* thresholds for these two binding sites did not matter). When this condition was violated either lysis alone or both lysis and lysogeny were altered and the two modified attractors described above were observed. Furthermore, when both *Cro* thresholds for *O*_*R*_2 were equal to the *Cro* thresholds for *O*_*L*_2 a particular variant of wild type lysis was detected. In some cases a modified lysogeny was observed while lysis did not deviate from wild type: it happened when the thresholds for *repressor* and *O*_*R*_3 were lower than the thresholds for *O*_*L*_1 and *repressor* regardless of the threshold ordering for *Cro*.

Now that we know crucial inequalities between binding site affinities we can suggest experiments that should yield modified behaviours. For example, we can exchange binding sites *O*_*R*_1,*O*_*R*_2,*O*_*R*_3 with sites *O*_*L*_1,*O*_*L*_2,*O*_*L*_3 on a phage *λ* genome thus swapping the affinities for these sites for both *Cro* and *repressor*. According to our analysis, this action should violate a necessary condition for binding site affinities and cause a modified lysis behaviour. We also suggest a subtler experiment of swapping *O*_*R*_2 and *O*_*L*_2 sites – a modified behaviour should arise after such a swap. Here, it is important that the *repressor* affinities for these sites can be freely exchanged together with *Cro* affinities.

To detect the modified attractors experimentally it is necessary to focus on their most significant differences from the wild type attractors. Such discrepancies should be observable regardless of precise concentrations and growth rates. A major difference between the wild type and modified lysis is the gradual degradation of the products of *Q, xis, cIII, N* and structural proteins to zero level within the mutant lysis (these proteins are not produced afterwards). A crucial discrepancy between the modified and wild type lysogeny is the consistent production of proteins coded by *cIII, xis* and *N* which in turn implies the presence of non-integrated phage particles for the lysogeny mutant. In addition to these observations it is necessary to ascertain whether the system is within the lysis or lysogeny mode. It can be done by detecting the proteins that are produced in the same way regardless of wild-type and modified behaviours. For example, the presence of *repressor* and *Cro* could be checked to make this conclusion. Otherwise the presence of, for example, *N* is ambiguous: it can indicate either the wild type lysis or modified lysogeny.

### Experimental detection of modified behaviours

The phage *λ* genome is very compact and economical – genes are densely packed and regulatory elements take comparatively little space. The *O*_*R*_ operator sequence is only 74 base pairs (bp) long, while the *O*_*L*_ operator covers approximately 100 bp sequence [30]. All *O*_*R*_ and *O*_*L*_ binding sites are of the same size, 17 bp long [31]. The space between *O*_*R*_1 and *O*_*R*_2 is 6 bp and the interval between *O*_*R*_2 and *O*_*R*_3 is 7 bp. The coding sequences of *cI* and *cro* genes are in physical proximity to *O*_*R*_ and *O*_*L*_ as are the promoters from which the transcription of either *cI* or *cro* is initiated [32]. Moreover, the proteins that can bind to sites within the same operator tend to form dimers and also interact with RNA polymerase [26]. The equivalent lengths of binding sites permit exchanging their sequences, but care must be taken to preserve crucial distances between nearby elements on a genome, otherwise the strength of regulatory interactions could be altered. Thus, swapping whole operators is more likely to be successful than swapping of individual sites.

The sequence variation and mutations of the binding sites belonging to *O*_*R*_ and *O*_*L*_, including the effects of mutations, have been described in [30, 33]. Of particular interest to us are the mutations that inactivate *O*_*R*_1 site so that *repressor* cannot bind to it. It has been observed experimentally that this type of inactivation leads to an increased affinity of *repressor* for *O*_*R*_3 coupled with a decreased affinity for *O*_*R*_2 in comparison with a wild type phage *λ*. This effect is due to *repressor* dimers occupying either *O*_*R*_1 and *O*_*R*_2 or *O*_*R*_2 and *O*_*R*_3 sites simultaneously. If the altered affinity for *O*_*R*_2 is smaller than the affinity for *O*_*L*_2, then such a mutant could validate our hypotheses.

## Discussion

Hybrid system models, such as the one introduced by us, close the gap between Boolean networks and differential equation models. Hybrid models allow to represent the dynamics of concentration changes without requiring detailed knowledge of kinetic parameters. Several extensions of Petri Nets also incorporate both continuous concentration changes and discrete parameters, among them a Petri Net formalism with stochastic time delays [34] and Hybrid Petri Nets having two types of places and transitions, discrete and continuous [35].

A disadvantage of many hybrid system models is the lack of mathematical tools and software for their analysis. Here, we address both of these problems. We build a characteristic graph for a biological system and then analyse its topology – this method allows us to identify all attractors of a model. Such an analysis also provides crucial information for suggesting experiments that can distinguish between various steady states. The scalability of our approach will depend on the number of constraints for binding site affinity orderings – having more constraints can drastically reduce the size of a characteristic graph.

Process Hitting is one of the alternative techniques that can be used in modelling networks with partial knowledge of interactions between biological entities; this framework does not require the knowledge of quantitative parameters [36]. The authors propose a method for inferring positive and negative influences of other genes on the expression of a particular gene (some influences can remain ambiguous). They represent the more complex logical functions that govern gene expression as constellations of several components, possibly involving time delays between their activation. In contrast, we propose to model each control function as one item. They enumerate all feasible parameterisations of a biological network – the number of these is exponential in the number of regulators of a network component, but some parameterisations can be ruled out by using biological constraints. This approach is similar to our way of constraining threshold orderings. Process Hitting was used to infer 40 parameters out of 195 for an ERBB receptor-regulated G1/S transition model with 20 components [37] and 77 out of 143 for a T-cell receptor model with 40 components [38].

Process Hitting framework has been augmented with time delays that model production and degradation rates of proteins [39]. The authors use linear functions with unknown gradients to model changes in concentrations between each pair of thresholds. They obtain ranges of delay parameters that allow moving to a certain successive state. From this information necessary conditions for reaching attractors can be derived. In contrast, we do not require the assumption of linearity of growth/degradation functions in our method of attractor analysis. Their framework was applied to the phage *λ* network described in [7] and a model of ERBB Receptor-regulated G1/S transition involved in the breast cancer.

Our proposed modelling framework has several similarities with asynchronous automata [40]. Within the latter approach logical functions determine whether a protein is synthesised or not; the expression level of a gene depends on whether proteins are bound or not bound to binding sites. Their model incorporates binding thresholds for pairs of proteins and binding sites (one threshold per pair). All thresholds have to be linearly ordered and ambiguities within an ordering are not allowed. For each expression level of a gene, differential equations describe the growth and degradation dynamics of the protein it produces.

The authors of [40] propose to draw a graph of possible sequences of states for a gene regulatory network. A state is determined by the positions of protein concentrations relative to thresholds. They detect attractors by analysing cycles in a state graph and determining which of these cycles and under what conditions are consistently up-regulating or down-regulating.

There are some important differences between our approach and asynchronous automata. They had to create separate states that correspond to protein concentrations being equal to threshold values – we do not need such a device, because our association and dissociation thresholds are distinct for each pair of a protein and a binding site. As a consequence we do not have to computationally consider all combinations of additional intermediate states.

The method in [40] requires that the relative protein concentration with respect to its binding thresholds coincides with the expression level of its producing gene. It means that both growth and degradation rates have to be faster for larger protein concentrations. We do not make such an assumption and instead allow the expression level of a gene to vary irrespective of the concentration range of the protein it produces. They rely on a coupling of differential equations and logical expressions in their attractor analysis, while we do not make assumptions about the types of differential equations for various expression levels.

Asynchronous automata have been used to analyse the phage *λ* gene regulatory network [7]. The models presented in [7] are simpler than our proposed model: the first incorporates only *cI* and *cro* together with their influences on other genes; the second model involves *cI*, *cro*, *cII* and *N*. None of these models allows to capture crucial differences between wild type and modified attractors which we describe here.

We incorporated various biological constraints that have been demonstrated experimentally in our model. We took into account the six conditions of the second model from [7]. In addition we included the negative loop of *cro* on itself, which prevents the products of *cro*, *cII*, *O* and *P* from rapid multiplication during replication, and incorporated the negative control by *repressor* of its own gene. Both *O*_*R*_ and *O*_*L*_ cascades were included in the second model from [7] and four binding thresholds were distinguished for both *repressor* and *Cro*. In comparison we considered six threshold pairs for each of these proteins. A particular order of *O*_*L*_ and *O*_*R*_ affinities was assumed in the earlier paper. In contrast, we checked a wide range of orderings to discover the essential conditions that determine the known phage *λ* behaviours. They did not include integration and excision in the model, while we modelled both *xis* and *int* and were able to detect alterations of integration and excision within modified lysis.

We detected only two stable behaviours for any phage *λ* variant. The authors of [7] arrived at the same conclusion, but they first detected two steady states and one stable cycle and only then deduced that just two stable behaviours are feasible. Our approach did not require discarding steady states on the basis of differential equation stable point analysis – instead we immediately determined attractors from the classification of strongly connected components.

A range of phage *λ* variants has been studied both experimentally and also by different modelling approaches. In [41] only the *O*_*R*_ cascade was modelled while *O*_*L*_ was not considered. Three different mutants were studied by inducing mutations either within *O*_*R*_1 or *O*_*R*_3 or in both. Identical binding affinities for *O*_*R*_1 and *O*_*R*_3 yielded stable lysogeny and a fully functional genetic switch. However, the phage in which *O*_*R*_1 and *O*_*R*_3 were switched could not lysogenise and formed only tiny plaques. The effects of these mutations are consistent with the predictions of our model – we obtain the wild type lysis and lysogeny whenever we do not contradict the mutual affinity ordering for *O*_*R*_ sites and leave the *O*_*L*_ cascade intact. Equal affinities are covered in our characteristic graphs as special cases where the transition time from one state to another is zero. The model presented in [42, 43] is simpler than our model – it includes only the *O*_*R*_ cascade, but not the *O*_*L*_ cascade; they model the changes in *repressor* and *Cro* amounts by differential equations, but omit an important influence of *CII* on the *λ* switch. They considered two mutants which make the lysogenic state unstable in nature – only lysis attractors were obtained under these conditions. These results do not capture the different types of lysis we have identified.

Our analysis of a phage *λ* model revealed a surprising robustness of this biological network. We explored all orderings not contradicting the chief requirements for two binding site cascades, *O*_*R*_1, *O*_*R*_2, *O*_*R*_3 and *O*_*L*_1, *O*_*L*_2, *O*_*L*_3. Each ordering yielded exactly two different attractors which could be easily mapped to lysis and lysogeny behaviours. We discovered only two modified versions of lysis and lysogeny behaviours. This degree of robustness is by no means guaranteed for any network. For example, the small network in Fig. 1 has a characteristic graph of less than ten vertices for any binding affinity ordering, yet we can obtain many different attractors.

Our method of qualitative analysis can be useful in deciding which models of a biological system are valid. Several alternative models can be considered and their stable behaviours identified: it is easy to check whether the predicted behaviours correspond to experimentally observable phenotypes. It is possible to use various properties of an attractor in a characteristic graph for detecting this particular behaviour experimentally, for example, consistent absence or presence of proteins can be tested.

## Conclusions

We propose a novel and computationally efficient method for analysing gene regulatory networks and discovering all stable behaviours of a network. It does not require precise information on quantitative parameters such as binding affinities and the characteristics of protein production and degradation. Our method is also applicable when the knowledge of the mutual ordering of binding site affinities is incomplete. We provide a software that implements the algorithm of attractor discovery.

We built a model of phage *λ* and were able to prove that this biological system can exhibit only two different stable behaviours given the main constraints of the *λ* switch. These behaviours correspond to lysis and lysogeny. According to our predictions, two versions of both lysis and lysogeny are possible, a wild type and a modified behaviour. We derived sufficient conditions for observing the wild type behaviour. We also suggested several experiments for detecting modified behaviours that correspond to altered binding site affinity orderings.

The stable behaviours of phage *λ* turned out to be almost entirely determined by the network topology and unaffected by exact quantitative parameters. Nevertheless, sometimes the relationships between the growth and degradation rates of various proteins fine tuned a behaviour within an attractor. It would be of interest to include the inequalities between different rates in network analysis – this could potentially offer a more nuanced understanding of stable behaviours and narrow down the set of feasible behaviours.

Finally, an automatic discovery of necessary conditions for a particular behaviour to occur is of great importance. This would help in deriving experimentally verifiable hypotheses and yield information about the robustness and modularity of a network.

## Additional file

Additional file 1 Software package implementing our proposed method of attractor analysis. It contains source files, user manual and the phage *λ* model described in this manuscript. Following subsections describe files from the package. ModelDescription.txt: Definition of the phage *λ* model that is analysed within this paper. ModelConstraints.txt: File that specifies partial constraints for the orderings of binding site affinities. Here, the constraints are applicable to our phage *λ* model. HSM_graph_analysis.cpp: The main component of the software that identifies all feasible states of a system. HSM_graph_analysis.h: The second component of the software for graph analysis. It is a C++ header file which contains definitions of classes and data structures. HSM_instructions.pdf: Instructions for compiling and running the software that constructs a graph describing all possible states of a system. Formats of input and output files are described as well. Thr.txt: File containing the orderings of binding site affinities for all proteins. ExtractStates.pl: Perl script that extracts all stable states from a state transition graph. MergeStates.pl: Perl script that characterises and summarises stable states. It aggregates the information about stable states for several state graphs. AnalyseStates.pl: Perl script that characterises stable states of a system by describing the feasible behaviours within them. ThresholdOrderings.pl: Perl script that generates all linear orderings of binding site thresholds that are consistent with a set of constraints defining partially known orderings. (ZIP 84 kb)

## Data Availability

All data generated or analysed during this study are included in this published article and its supplementary information files.
