# Supplementary material for: Dynamics of gene regulatory networks and their dependence on network topology and quantitative parameters – the case of phage λ
Source: BMC Bioinformatics. 2019 May 31;20:296. doi: 10.1186/s12859-019-2909-z (PMC6544977; doi:10.1186/s12859-019-2909-z)
Supplement: Supplementary file 1 — Software package implementing our proposed method of attractor analysis. It contains source files, user manual and the phage λ model described in this manuscript. Following subsections describe files from the package. ModelDescription.txt: Definition of the phage λ model that is analysed within this paper. ModelConstraints.txt: File that specifies partial constraints for the orderings of binding site affinities. Here, the constraints are applicable to our phage λ model. HSM_graph_analysis.cpp: The main component of the software that identifies all feasible states of a system. HSM_graph_analysis.h: The second component of the software for graph analysis. It is a C++ header file which contains definitions of classes and data structures. HSM_instructions.pdf: Instructions for compiling and running the software that constructs a graph describing all possible states of a system. Formats of input and output files are described as well. Thr.txt: File containing the orderings of binding site affinities for all proteins. ExtractStates.pl: Perl script that extracts all stable states from a state transition graph. MergeStates.pl: Perl script that characterises and summarises stable states. It aggregates the information about stable states for several state graphs. AnalyseStates.pl: Perl script that characterises stable states of a system by describing the feasible behaviours within them. ThresholdOrderings.pl: Perl script that generates all linear orderings of binding site thresholds that are consistent with a set of constraints defining partially known orderings. (ZIP 84 kb) [file 12859_2019_2909_MOESM1_ESM.zip › HSM/HSM_instructions.pdf]

HSM – a hybrid system based modeling and model analysis software

Dace Ruklisa

March 18, 2019

Contents

1 Introduction **1**

2 Constructing a state transition graph **2**

2.1 Model description 2

2.2 Orderings of binding thresholds 5

2.3 Output and visualisation of state transition graphs 5

3 Extracting of stable states from state graphs **6**

4 Determining characteristics of stable states **6**

5 Generating threshold orderings **8**

1 Introduction

This documentation describes an implementation of a hybrid system based modelling tool that is tailored for analysing gene regulatory networks. The main algorithm encompassed in this software constructs a state transition graph that covers all possible states of a biological system. Our method automatically identifies all stable states of a system. The algorithm of graph construction has been first introduced in [2]. The format that is used for describing models is based on the finite state linear model defined in [1], albeit here information about quantitative parameters is omitted and functions are monotonous instead of linear.

The main component of this package is a C++ software captured in two files: HSM_graph_analysis.cpp and HSM_graph_analysis.h. This software has to be compiled before use; Gnu C++ compiler g++ can be used for this purpose among other options.

In addition several Perl scripts are provided for further analysis of stable states: ExtractStates.pl, MergeStates.pl, AnalyseStates.pl and ThresholdOrderings.pl.

The visualisation of state transition graphs relies on the Graphviz software that is open source. For more information about using Graphviz and for downloading it see <https://www.graphviz.org>.

2 Constructing a state transition graph

The algorithm that constructs a state transition graph is implemented in the HSM software which consists of two source files: HSM_graph_analysis.cpp and HSM_graph_analysis.h. To compile it with the g++ compiler, run the following commands:

```
g++ HSM_graph_analysis.cpp -o HSM.out
mv HSM.out HSM.exe
```

It is necessary to move to the directory where both files are stored beforehand.

To construct a state transition graph run the following command:

```
/myPath/HSM.exe /myPathToModels/ModelDescription.txt
  /myPathToThresholds/Thresholds/Thr.txt /myPathToGraphs/Graph.txt
```

It is necessary to provide three parameters to the algorithm. The first is the file containing a model description (/myPathToModels/ModelDescription.txt); the second is the file with fully specified threshold orderings for all proteins that have binding thresholds (/myPathToThresholds/Thresholds/Thr.txt); the last is the name of the state graph file which will either be created anew or rewritten (/myPathToStates/States.txt).

2.1 Model description

An example of a model description file is included in the package (ModelDescription.txt). This particular file describes a phage λ model. The first section of such a file defines all proteins that are modelled and consists of instructions

```
SUBSTANCE Struc
```

where the keyword ‘SUBSTANCE’ is followed by a protein name.

The second section defines all binding sites. The description of a site begins by an instruction

```
BINDINGSITE bN
```

where a site name is written after the keyword ‘BINDINGSITE’. It is followed by the list of all proteins that can bind to this site; a protein is attached to a site via the instruction

```
BINDINGFACTOR CI
```

where ‘BINDINGFACTOR’ precedes a protein name. Each name must correspond to a definition of a protein in the first section.

The third section describes all promoters. A promoter definition starts with a line

```
PROMOTER PME
```

where ‘PROMOTER’ is followed by a promoter name. Subsequent lines specify all genes that are regulated by this promoter. A gene is attached to a promoter by the instruction

```
GENE CII
```

where ‘GENE’ is followed by a gene name. Here, gene names are assumed to be identical to protein names. Thus, each name has to correspond to a definition in the first section.

Each promoter has exactly one control function that is attached to it by the command

```
CONTROLFUNC 3-3-3-2-PR
```

where ‘CONTROLFUNC’ is followed by a function name.

A promoter definition is finalised with a list of binding sites belonging to this promoter. These binding sites correspond to the input variables for the control function of this promoter. The order in which sites are introduced is important as it corresponds to the list of control function arguments. Each binding site is defined by a command

```
BINDINGSITE bOR3
```

where ‘BINDINGSITE’ is followed by a site name. This name must correspond to a binding site definition in the second section.

Control functions are specified in the last section of a model file. Each definition starts with the line

```
CONTROLFUNCTION
```

and ends with the line

```
END
```

The second line of a control function description contains its name, for example,

```
2-3-3-3-2-PINT
```

This name must correspond to some function name within a promoter definition, otherwise the function will not be successfully assigned to a promoter. The third line of a description lists all binding sites whose states serve as arguments for this function:

```
bCII-2 bOL3 bOL2 bOL1
```

The order of the sites has to be identical to the site sequence within a promoter definition that involves this function. The function itself is defined via a table of inputs and outputs comprising lines such as

```
0 0 1 0 0
```

Here, the last number in a line specifies the expression level of a gene that corresponds to a combination of binding site states which precede it. The states have to be listed according to the order of sites specified in the promoter definition and in the function header. Each state is denoted either by ‘0’ meaning an unoccupied site or by a number greater than zero meaning that the site is occupied by a particular protein. Proteins are numbered according to the sequence of proteins within the binding site definition. For example, if the site definition is

```
BINDINGSITE bOL2
BINDINGFACTOR CI
BINDINGFACTOR CRO
```

then ‘1’ in the function table means that this site is occupied by CI, while ‘2’ means occupation by CRO.

2.2 Orderings of binding thresholds

This package contains an example file of threshold orders (Thr.txt). Each line of such a file specifies an ordering of all binding thresholds for a single protein as in the following example

```
ORDER CRO = bOL3.dis bOL3.as bOR3.dis bOR3.as bOL2.dis bOL2.as
  bOR2.dis bOR2.as bOR1.dis bOR1.as bOL1.dis bOL1.as
```

The keyword 'ORDER' starts each line of a threshold file and is followed by a protein name. The protein name must correspond to a definition of a substance within a model file. After '=' all thresholds are listed starting from the lowest to the highest. Each threshold is identified by a binding site name concatenated with either '.as' or '.dis' indicating an association or a dissociation threshold respectively.

2.3 Output and visualisation of state transition graphs

A state transition graph that is generated by the HSM tool is stored in a format suitable for visualising with Graphviz. The extension of the output file has to be changed to '.dot' before it is read by Graphviz. To visualise such a graph, launch Graphviz and then go to the 'File' section within the main toolbar and select 'Open...' from the menu. Afterwards find the chosen state transition graph file. The graph will be shown on a screen; it can be saved to a file by selecting 'Export...' from the 'File' section within the main toolbar.

Rectangles in a visualised graph correspond to closely connected components. Each rectangle comprises one or more states (labelled vertices). Components are coloured. Yellow colour denotes a transient component having a progress indicator. The latter is a protein whose concentration is guaranteed to either grow or decrease beyond the boundaries of component's states. Thus, any such component will inevitably be left after a finite time. Blue and magenta colours denote permanent components that neither have any transitions leading out of them nor any progress indicators. A magenta component consists of a single cycle. A blue component comprises at least one state that leads towards different states; it has several cycles – the sequence of states visited within this component will be determined by mutual relationships between quantitative parameters. All attractors are parts of permanent components.

Each state is marked by expression levels of all genes. The name of a gene is followed by either 'u' or 'd' meaning that the corresponding protein respectively grows or decreases within this state. A state is uniquely identified by all binding site states which are listed after the 'state' keyword. A state of a site is described by a binding site name followed by a number. Here,

‘0’ indicates an empty site and numbers greater than zero imply an occupied site. Proteins are numbered according to the sequence of proteins within the binding site definition. For example, if the site definition is

```
BINDINGSITE bOL2
BINDINGFACTOR CI
BINDINGFACTOR CRO
```

then state ‘1’ means that this site is occupied by CI, while ‘2’ means occupation by CRO.

Each transition is labelled by the condition that triggers it. For example, if a label is ‘Q >= bQ.as’ then the protein Q grows within the source state and has to reach the association threshold bQ.as for this transition to take place.

3 Extracting of stable states from state graphs

It is possible to extract all stable states from a state transition graph and to view them separately. It can be done with the Perl script ExtractStates.pl by executing the command

```
perl ExtractStates.pl "/myPathToGraphs/Graph.txt"
  ">/myPathToStates/States.txt"
```

It is necessary to move to the directory where the script is stored beforehand.

This software requires providing two parameters (it is recommended to enclose each argument by quotes). The first is the file name of a state transition graph. The second is the name of an output file which will be either created anew of rewritten – the stable states of the state transition graph will be written there. Stable states correspond to permanent components. For more information about various types of components and attractors see the section Output and visualisation of state transition graphs.

Stable states are written in a format suitable for visualisation with Graphviz. For further information about the output and visualisation see the section Output and visualisation of state transition graphs.

4 Determining characteristics of stable states

Stable states can be characterised and summarised by the Perl script MergeStates.pl. This script obtains information about the number of stable states

within the graph for each threshold ordering. It writes distinct stable states found for various threshold orderings. The script is particularly useful when several threshold orderings have been analysed and results have to be aggregated. It can be run by the command

```
perl MergeStates.pl "/myPathToStates/States_" 1 576
  ">/myPathToStateSummaries/AllStates.txt"
  ">/myPathToStateCounts/StateCounts.txt"
  ">/myPathToStateThresholds/StateThresholds.txt"
```

It is necessary to move to the directory where the script is stored beforehand.

All six arguments for the script are compulsory and do not have default values. The first is the path to the files containing stable states together with the prefix shared by all file names. The next two arguments are numbers that specify the range of names of stable state files. The script concatenates the file prefix with numbers in such a way that the file names /myPathToStates/States_1.txt , ..., /myPathToStates/States_576.txt are obtained by executing the above command. The fourth argument is the output file for distinct stable states from all stable state files. Stable states are written in a format suitable for visualisation with Graphviz. For more details about the output and the visualisation see the section Output and visualisation of state transition graphs. The fifth argument is the name of the output file where stable state counts are written. The first column in this file contains the numbers of stable state files, while the second column records the stable state counts for respective files.

The sixth argument is the output file where threshold orderings are mapped to stable states. The first column contains the numbers of stable states (the numbers correspond to the order of different stable states in the file that is given as the fourth argument). The second column lists all stable state files where a particular stable state can be found. Both columns are separated by ':'.

Stable states can be characterised further by determining the number of states within them and also the observable behaviour of proteins. This can be done by the command

```
perl AnalyseStates.pl "/myPathToStateSummaries/AllStates.txt"
  ">/myPathToStateDescr/StateDescriptions.txt"
```

It is necessary to move to the directory where the script AnalyseStates.pl is stored beforehand.

The first argument is the file containing permanent components – these can be either the components extracted from a state transition graph or the ag-

gregated components from several graphs that have been collected by MergeStates.pl. The second argument is the output file where component descriptions are written.

A description of a stable state begins with a component number and is immediately followed by the number of states within the component as in

```
component 1 vertices 2
Struc -2 | Q -2 | CII -2 | P -2 | O -2 | CRO -2 | CI 0 | INT -2 |
XIS -2 | CIII -2 | N -2
```

The second line describes the observable behaviour for each protein. A protein name is followed by the integer that denotes the difference between the amount of states in which this protein is increasing and the count of states where this protein is decreasing. For example, '0' means that a protein is increasing in half of the states within the component and decreasing in the other half. Similarly, '-2' implies that the protein is consistently decreasing in all states because the total number of states within this component is two.

5 Generating threshold orderings

Another software component, ThresholdOrderings.pl, generates all complete orderings of protein thresholds that are consistent with a set of constraints defining partially known orderings. The script can be run by the command

```
perl ThresholdOrderings.pl
  "/myPathToConstraints/ModelConstraints.txt"
  ">/myPathToThresholds/Thr" 1
```

The first argument is the file containing partial constraints for threshold orderings. The second argument is the prefix of all threshold ordering files that will be created by the script. Output files will have names /myPathToThresholds/Thr_1.txt etc.. The final argument is the number of the first threshold ordering; consecutive numbers will be assigned to subsequent orderings starting from the initial number.

Let us consider the format of a threshold constraint file. An example file is attached to this package (ModelConstraints.txt). The first section defines all proteins included in the model and contains lines of the form

```
SUBSTANCE Struc
```

where the keyword 'SUBSTANCE' is followed by a protein name. The second section defines all binding sites. A description of a site begins by an instruction

```
BINDINGSITE bN
```

where a site name is written after the keyword 'BINDINGSITE'. The list of all proteins that can bind to this site follows. A protein is attached to a site via the instruction

```
BINDINGFACTOR CI
```

where 'BINDINGFACTOR' precedes a protein name. Each name must correspond to a definition of a protein in the first section.

The third section comprises partial orderings of binding site affinities. Known inequalities between affinities are specified by lines such as

```
ORDER CI = bOR1.dis < bOR1.as < bOR2.dis < bOR2.as < bOR3.dis <
bOR3.as
```

Here, the keyword 'ORDER' is followed by a protein name – all thresholds mentioned in this line correspond to this protein. Each protein name must correspond to a definition of a protein in the first section. After '=' a sequence of inequalities is written, where thresholds are arranged in an increasing order and separated by '<'. Each threshold is identified by a binding site name concatenated with either '.as' or '.dis' indicating an association or a dissociation threshold respectively. The site name must correspond to a binding site definition in the second section. The protein mentioned at the beginning of the line has to be listed as a binding factor for all sites within this line.

A protein can have several lines of partial orderings and even no lines at all.

The partial orderings must incorporate the condition that for each binding site and protein pair the association threshold has to be greater than the dissociation threshold.

Another type of instruction specifies that no other thresholds can be located between the association and the dissociation threshold for a certain pair of a binding site and a protein. Such an instruction is written as

```
CLOSE_THRESHOLDS CRO = bOR1 bOR2 bOR3 bOL1 bOL2 bOL3
```

Here, the keyword 'CLOSE_THRESHOLDS' is followed by a protein name. Each name must correspond to a definition of a protein in the first section. After '=' one or more binding sites are listed – for each of these sites and the corresponding protein both thresholds will be close in any generated orderings. The site name must correspond to a binding site definition in the second section. The protein mentioned at the beginning of the line has to be listed as a binding factor for all sites within this line.

References

[1] Brazma A, Schlitt T. Reverse engineering of gene regulatory networks: a finite state linear model. Genome Biology. 2003 April;4:P5 DOI: 10.1186/gb-2003-4-6-p5.

[2] Brazma A, Cerans K, Ruklisa D, Schlitt T, Viksna J. HSM – a hybrid system based approach for modelling intracellular networks. Gene. 2013 April;518(1):70–77 doi:10.1016/j.gene.2012.11.084.
